# Supplementary material for: Splice-Junction-Based Mapping of Alternative Isoforms in the Human Proteome
Source: Cell Rep. Author manuscript; Available in PMC 2020 Jan 15. (PMC6961840; doi:10.1016/j.celrep.2019.11.026)
Supplement: 3 [file NIHMS1546469-supplement-3.zip › DF2/PXD0010154/Ovary-207-O43291-ERSIHENATGDLATSRNAA.pdf]

A

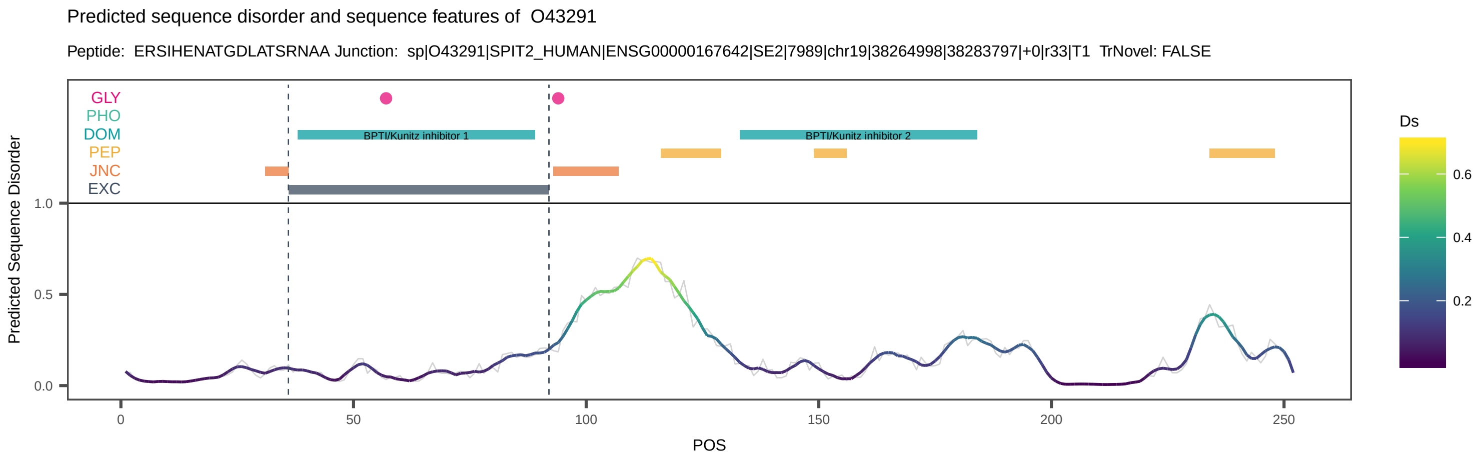

Predicted sequence disorder and sequence features of O43291
Peptide: ERSIHENATGDLATSRNAA Junction: sp|O43291|SPIT2_HUMAN|ENSG00000167642|SE2|7989|chr19|38264998|38283797|+0|r33|T1 TrNovel: FALSE
GLY
PHO
DOM
PEP
JNC
EXC
BPTI/Kunitz inhibitor 1
BPTI/Kunitz inhibitor 2
Predicted Sequence Disorder
1.0
0.5
0.0
0
50
100
150
200
250
POS
Ds
0.6
0.4
0.2

B

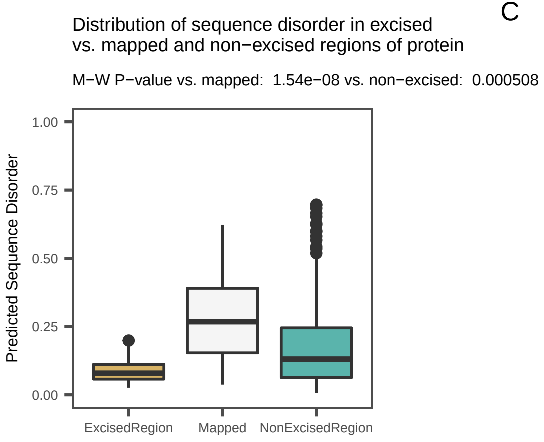

Distribution of sequence disorder in excised
vs. mapped and non−excised regions of protein
M−W P−value vs. mapped: 1.54e−08 vs. non−excised: 0.000508
Predicted Sequence Disorder
1.00
0.75
0.50
0.25
0.00
ExcisedRegion
Mapped
NonExcisedRegion

C
